# Liver transplantation as a new treatment option for perihilar cholangiocarcinoma and colorectal liver metastases: a review

**DOI:** 10.1007/s10147-025-02820-3

**Published:** 2025-07-05

**Authors:** Ken Fukumitsu, Takashi Ito, Shoichi Kageyama, Satoshi Ogiso, Takayuki Anazawa, Kazuyuki Nagai, Yoichiro Uchida, Takamichi Ishii, Etsuro Hatano

**Affiliations:** 1 Department of Surgery, Graduate School of Medicine, Kyoto University, 54 Kawahara-Cho, Shogoin, Sakyo-Ku, Kyoto, Kyoto 606-8507 Japan; 2 Department of Surgery, Kyoto Katsura Hospital, 17 Yamadahirao-Cho, Nishigyo-Ku, Kyoto, Kyoto 615-8256 Japan

**Keywords:** Liver transplantation, Cholangiocarcinoma, Colorectal liver metastasis

## Abstract

Liver transplantation (LT) has become widespread in recent years owing to advances in the elucidation of its pathogenesis, surgical procedures, and perioperative management. Historically, LT was only performed in patients with end-stage liver disease and certain malignancies, such as hepatocellular carcinoma, but its indications have recently been expanded to include unresectable perihilar cholangiocarcinoma (pCCA) and colorectal liver metastases (CRLM). In this review, we discuss the current status and future prospects of LT for these expanded indications. Perihilar cholangiocarcinoma and colorectal liver metastasis have poor prognoses if they cannot be surgically resected. For non-resectable pCCA, neoadjuvant chemoradiotherapy followed by LT has demonstrated improved survival, particularly under the Mayo Clinic protocol. Furthermore, LT for CRLM has received renewed interest following encouraging results from a Norwegian group showing a 5-year survival rate of > 80% with strict selection criteria. A recent randomized controlled trial further validated LT with chemotherapy as a promising option, demonstrating a significant survival advantage over chemotherapy alone. Both achieved favorable outcomes by implementing strict patient selection criteria and integrating LT as part of a multidisciplinary treatment approach that includes chemotherapy and radiation therapy. As transplant oncology continues to evolve, a multidisciplinary approach integrating transplant surgery, oncology, and hepatology is crucial for refining LT protocols for non-resectable pCCA and CRLM. Ongoing clinical trials and translational research are key to defining the role of LT in this expanding field, potentially establishing it as a standard therapy for selected cases of advanced hepatic malignancies.

## Introduction

Liver transplantation, first performed by Starzl in Denver, Colorado in 1963, has become a globally accepted definitive treatment for end-stage liver disease. This has been driven by advances in surgical techniques, expanding knowledge, and the development of novel immunosuppressive agents. Although its efficacy is well-established for end-stage liver cirrhosis, metabolic diseases, and hepatocellular carcinoma, indications for liver transplantation are expanding to include unresectable cholangiocarcinoma and colorectal liver metastases. This review aimed to provide an overview of the current status and future prospects of liver transplantation for these expanded indications.

## Transplant oncology

Oncology and transplantation medicine have been regarded as distinct disciplines. However, the integration of these two fields has given rise to the concept of transplant oncology, which was first proposed in 2014 as a novel approach to advance the treatment and research of refractory and advanced malignancies [1]. In 2021, transplant oncology was formally defined as “the application of transplant medicine and transplant surgery with the aim of improving the prognosis and quality of life of cancer patients.” Advances in chemotherapy, immunosuppressive therapies, and surgical techniques have enabled previously unfeasible treatments, thereby expanding the therapeutic options available to patients.

## Liver transplantation for perihilar cholangiocarcinoma

### Background and historical overview

Perihilar cholangiocarcinoma is a rare malignant tumor of the hepatic hilum. While complete resection offers the best prognosis, surgical resection is often challenging because of frequent tumor invasion into critical vascular structures [2]. Since the 1980s, liver transplantation has been explored as a treatment for unresectable perihilar cholangiocarcinoma. In 1988, a series of 18 cases were reported in Germany; however, four patients died within 60 days, and data on long-term outcomes were limited [3]. Further reports on long-term survival were published in 1996 in Germany and in 1998 in the United States, both of which indicated a 5-year survival rate of approximately 25% [4, 5]. By the 2000s, the results remained unfavorable [6, 7], and liver transplantation for cholangiocarcinoma was regarded as contraindicated.

Subsequently, improvements in treatment protocols were made at the Mayo Clinic. In 2005, the introduction of a comprehensive protocol involving stringent patient selection criteria, preoperative radiation therapy, and chemotherapy yielded promising results [8]. A multicenter study involving 12 institutions in the United States in 2012 showed a significant improvement in the 5-year survival rate (53%) [9]. Consequently, liver transplantation has become recognized as an acceptable treatment modality, not only in the United States but also globally, and is widely practiced. Given that liver transplantation protocols and chemotherapy regimens vary by country and region, adaptations have been made to the Mayo protocol (Table 1).

**Table 1:** Variation of Mayo protocol

Institute	Radiotherapy		Chemotherapy	
	External beam	Brachytherapy (Iridium 192)		Maintenance until Tx
Mayo clinic (US) [8]	45 Gy (1.5 Gy/day, 30 fr)	20–30 Gy	5-FU	Capecitabine
UCLA (US) [10]	40 Gy (5 fr)	–	Gemcitabine, cisplatin	Gemcitabine and cisplatin
Toronto (Canada) [11]	55-75 Gy (1.5 Gy/day for 4–5 weeks)	Selected patient	Capecitabine	Gemcitabine and Cisplatin
St Vincent's (Irland) [12]	45–55 Gy (usually 50 Gy, 2 Gy/day, 5 weeks)	7.5 Gy	5-FU	Capecitabine
Kyoto [13], Kumamoto (Japan)	50-56 Gy (Kyoto) 45-50 Gy (Kumamoto)	–	Gemcitabine, cisplatin, tegafur gimeraciloteracil potassium	–

For example, in Japan, where the number of deceased liver transplants is low, living donors are used instead of deceased donors, and chemotherapy is based on a regimen of gemcitabine and cisplatin rather than fluorouracil (UMIN000033348, jRCT1070220052).

### Definition of unresectability

Liver resection for perihilar cholangiocarcinoma is a complex surgical procedure, and the determination of its unresectability can vary across institutions. Some facilities may consider Bismuth type IV tumors unresectable.

On the other hand, there have been reports suggesting that bile duct cancers with invasion extending to the umbilical portion—previously deemed unresectable—may be amenable to R0 resection through the Meso-Rex shunt approach [14–16]. A clinical trial initiated by Kyoto University in 2021defined criteria for indications of unresectability; (i) Insufficient remnant liver function: (ii) Cases in which vascular invasion precludes adequate blood supply to the remnant liver: (iii) Extensive horizontal tumor spread beyond the feasible bile duct transection margin: (iv) Cholangiocarcinoma of undetermined localization associated with primary sclerosing cholangitis (PSC) [13].

The trial has now been completed and subsequently succeeded by a nationwide multicenter trial led by Kumamoto University (jRCT1070220052). This trial, conducted with governmental support, aims to establish a new standard of care for Japan. Its forthcoming outcomes are highly anticipated. Distant or intrahepatic metastases are contraindications for liver transplantation. Prior to transplantation, staging surgery should be performed to confirm the absence of malignant findings. Additionally, if tumor extension toward the pancreas is observed, pancreaticoduodenectomy should be performed with liver transplantation, provided that complete resection of all tumor lesions is achievable.

### Differences in protocols between deceased and living donors

In Western countries, deceased donors are the standard source of organ donation; however, due to religious and cultural factors, there is a shortage of deceased donors in certain countries. In such countries, living donors are most commonly used. In Japan, living donor liver transplantation (LDLT) is the standard procedure performed under a protocol distinct from that used for deceased donors. A key disadvantage of LDLT is the smaller graft size; however, it offers the advantage of scheduling the surgical date in advance. In the case of deceased donor liver transplantation (DDLT), chemotherapy must be continued until the donor becomes available after staging surgery has confirmed its suitability for liver transplantation. In contrast, LDLT allows for the scheduling of surgery once chemotherapy has been administered and the effectiveness of the treatment has been assessed. Radiotherapy is administered on the basis of the calculated timeline from the scheduled liver transplantation date, after which the liver transplant is performed.

The surgical techniques employed for the two types of transplantation differ significantly. In this case, radiation is applied to the hepatic hilum, and to perform lymph node dissection around the hepatic hilum, the hepatoduodenal ligament is resected as a single block. Additionally, owing to the limitations of available vascular grafts, reconstruction of the portal vein and hepatic artery requires careful consideration. For portal vein reconstruction, a long graft is often required to compensate for the defect at the hepatic hilum. Additionally, arterial reconstruction is necessary for hepatic artery anastomosis. These technical complexities increase the difficulty of the procedure and are associated with a higher incidence of complications than DDLT [17].

### Potential expansion of the indications for liver transplantation in resectable cases

A study using data from 10 American institutions reported on cases of liver transplantation performed following preoperative adjuvant chemotherapy for “resectable” perihilar cholangiocarcinoma [18]. A comparison between resection and liver transplantation for resectable cholangiocarcinoma demonstrated that the liver transplantation group had significantly better survival rates than the resection group (64% v.s. 18% in five-year overall survival). While liver transplantation has been suggested as a potential treatment option even for resectable cases, concerns have been raised regarding the lower survival rates in liver resection cases than in other reports [19, 20]. Therefore, further investigation is needed before expanding the indications for liver transplantation to include resectable cases.

## Unresectable colorectal liver metastases

### Background and previous developments

Colorectal cancer is considered a tumor for which a favorable prognosis can be expected if surgically resected, even in the presence of metastases to other organs. The main parenchymal organs that metastasize are the liver and lungs. However, hepatic metastasis is a prognostic factor in some cases. Hepatic resection is recommended for resectable cases, but when the disease is deemed unresectable and chemotherapy is administered, the long-term prognosis is poor. Various approaches have been explored for the treatment of liver metastases of unresectable colorectal cancer. With advancements in chemotherapy, response rates have improved, leading to an increased number of conversion surgeries in which initially unresectable cases become resectable [18]. Although the prognosis of patients undergoing conversion surgery is favorable, it remains poor for patients with unresectable or residual tumors. Even in cases where metastases are confined to the liver, patients may eventually die of liver failure. Despite progress in chemotherapy, the 5-year survival rate of patients with unresectable colorectal cancer with liver metastases remains below 10% [21–23].

Liver transplantation is an attractive treatment option for colorectal liver metastases limited to the liver. The use of liver transplantation for colorectal metastasis dates back to 1963 [24]. Of the first seven cases of liver transplantation, two patients had colorectal cancer liver metastases, one patient died on postoperative day 11 due to pneumonia and a liver abscess, and the other died on the same day as surgery due to intraoperative bleeding. Subsequent attempts at various institutions showed poor outcomes, with a 5-year survival rate ranging from 12–21% and a 30-day mortality rate of 11–30% [25]. According to the report from the University of Cincinnati in 1991 [26], among 41 patients who underwent liver transplantation for metastatic liver tumors, 10 cases involved colorectal liver metastases (CRLM). The five-year overall survival rate was 12%. One-third of the patients died within 30 days postoperatively, and two-thirds experienced recurrence before the end of the study period. Postoperative management included high-dose cyclophosphamide and total body irradiation, as well as simultaneous resection of peritoneal metastases during transplantation. According to the report from the University of Vienna [27], liver transplantation was performed in 10 patients with CRLM. The five-year survival rate was 21%, but the 30-day mortality rate was approximately 30%, suggesting issues related to technical quality and immunosuppressive therapy. Taken together, these problems are inappropriate patient selection criteria, surgical techniques, and immature therapeutic strategies involving immunosuppressive agents. These results are not considered acceptable; therefore, liver transplantation for colorectal liver metastasis is considered contraindicated.

In 2013, a Norwegian group reported promising results for liver transplantation in patients with unresectable colorectal liver metastasis, with 1-year, 3-year, and 5-year survival rates of 95%, 68%, and 60%, respectively [28]. While this pilot study, named SECA-I, included only 25 patients, the marked improvement in outcomes compared with those in previous reports was groundbreaking, although recurrence was observed in all patients within 2 years. In the SECA-I trial, the research group identified four prognostic factors: tumor size (< 5.5 cm), carcinoembryonic antigen (CEA) level (< 80 μg/L), waiting time (≥ 2 years), and response rate (partial response and stable disease). Hence, they implemented more stringent patient selection criteria for a new trial. In the new trial, SECA-II, the 1-year, 3-year, and 5-year survival rates were extended to 100%, 83%, and 83%, respectively, with 5-year recurrence-free survival rates of approximately 53%, 44%, and 35%, respectively [29]. Even among patients who experienced recurrence after transplantation, approximately 60% had no evidence of disease 5 years after transplantation. This trial highlighted that although recurrence occurs often after liver transplantation, many recurrent lesions are treatable, demonstrating an overall promising prognosis for these patients.

### Indications

#### Definition of unresectability

Even in cases initially deemed unresectable at the time of diagnosis, advancements in chemotherapy, along with technical innovations in liver resection have made it possible to achieve resectability in many cases. However, cases that remain unresectable are due to anatomical constraints that prevent sufficient remnant liver volume, raising concerns about potential liver failure, and hepatic dysfunction that impairs the function of the remnant liver, leading to concerns about liver failure. Anatomical constraints involve cases where multiple liver metastases are widely spread across both lobes of the liver, or when tumor invasion into major vascular structures makes it difficult to maintain blood flow to the remnant liver. In such cases, even if the liver function is adequate, sufficient remnant liver volume cannot be preserved. Hepatic dysfunction refers to situations in which chemotherapy-induced hepatic injury increases the risk of postoperative liver failure, leading to the case being deemed unresectable [30, 31]. Even in cases where chemotherapy is highly effective, if it cannot be continued due to liver dysfunction, it may represent a good indication for treatment.

#### Tumor criteria

Evaluation of the biological malignancy of a tumor is also crucial. In cases of highly biologically malignant tumors, even if the tumor can be surgically resected with sufficient margins, a high recurrence rate often leads to a poor prognosis. A group from Norway defined the Oslo score using the four aforementioned factors (tumor diameter > 5.5 cm, CEA > 80 ng/ml, Progressive Disease, < 2 years from primary surgery to liver transplantation) and used it as a prognostic predictor of liver transplantation outcomes. Additionally, the Fong score (lymphoid metastasis, disease-free interval < 12 months, number of hepatic tumors > 1, hepatic tumor size > 5 cm, CEA > 200 ng/ml) [32] has been shown to correlate with prognosis after liver transplantation. The Consensus Guidelines published by the International Hepato-Pancreato-Biliary Association in 2021 [33] discuss eligibility criteria from multiple perspectives. Generally, even if intrahepatic metastases are localized on imaging studies, the presence of microscopically undetectable extrahepatic metastases precludes liver transplantation as an appropriate treatment option.

Specifically, patients with progressive disease during chemotherapy, elevated tumor markers, lymphatic invasion (ly( +)), or lymph node metastasis (n( +)) in primary tumors were considered ineligible [34]. The analysis of factors determining recurrence or prognosis after transplantation remains an area of limited evidence; thus, further high-quality clinical research will be necessary to refine and determine appropriate eligibility criteria.

### Methods of evaluation

#### Overall survival and recurrence-free survival

Overall survival and recurrence-free survival are commonly used metrics to evaluate cancer treatment. However, in the context of liver transplantation for colorectal cancer with liver metastasis, although recurrence occurs at a certain frequency, many of these recurrences are treatable, and even in cases of recurrence, the prognosis is considered favorable [35]. Regarding treatment efficacy for recurrence after transplantation, a study investigating pulmonary metastasis recurrence following liver transplantation found that most pulmonary metastases grow slowly and often appear peripherally, making them resectable in many cases. The 5-year overall and recurrence-free survival rates after pulmonary metastasectomy were 51% and 39%, respectively, indicating that treatment following recurrence was highly effective [36, 37]. Therefore, it is more appropriate to use overall survival rather than recurrence-free survival as the primary evaluation metric after liver transplantation.

#### Other evaluation criteria

With the remarkable advancements in chemotherapy for colorectal cancer, long-term survival has become increasingly achievable. Overall survival and recurrence-free survival alone are no longer sufficient outcome measures. Given that chemotherapy alone rarely achieves a cure, patients continue treatment over an extended period until it becomes unfeasible due to diminishing efficacy or intolerable adverse effects. The necessity for frequent hospital visits imposes a significant time burden, while adverse effects place substantial strain on the body, leading to a profound decline in quality of life (QOL).

Although liver transplantation is a technically demanding and high-risk procedure, it has the potential to improve post-transplant QOL compared with the preoperative state. An increasing number of ongoing clinical trials worldwide have incorporated QOL as an essential evaluation parameter. The forthcoming publication of these results is highly anticipated as they may provide valuable insights into the broader benefits of liver transplantation in this patient population.

The economic implications of medical care will become increasingly important in the future. The advent of molecular targeted therapies and subsequent immunotherapies has led to increasing drug costs, and the prolonged administration of these high-priced treatments has become a significant societal concern due to the escalating financial burden on healthcare systems [38].

Although liver transplantation is an expensive medical intervention that requires lifelong immunosuppressive therapy, it is comparatively less costly than long-term chemotherapy or immunotherapy. Although Bjornelv et al. reported that chemotherapy is less costly than liver transplantation, considering the high expense of recent molecular targeted therapies and immune checkpoint inhibitors (ICIs), as well as their potential to prolong survival, it is conceivable that liver transplantation may become the more cost-effective option in the near future [39]. Moving forward, it is essential to evaluate the cost-effectiveness of continued chemotherapy versus liver transplantation to guide optimal clinical and economic decision-making.

### Current status of ongoing clinical trials

Various clinical trials are being conducted worldwide, and 23 have been conducted on the basis of publicly available information (Table 2). Most of these were single-arm studies with low evidence levels. Although the potential efficacy of liver transplantation has been recognized, concerns regarding organ availability have been prioritized. However, five randomized controlled trials have been conducted; the French TRANSMET trial (NCT02597348) reported results in 2024 [40, 41]. In this trial, the 5-year overall survival rates were 73.2% versus (vs.) 9.3% (*P* < 0.0001) in the per-protocol analysis and 56.6% vs. 12.6% (*P* = 0.0003) in the intention-to-treat analysis comparing the chemotherapy plus liver transplantation and chemotherapy-only groups.

**Table 2:** Ongoing Clinical Trials for Non-resectable Colorectal Liver Metastases

Design	Title	ID	Enrollment	Country	Brief description	Date	Status
RCT DDLT	SECA-II	NCT01479608	25	Norway		Apr. 2012–Dec. 2027	Recruiting
	TRANSMET	NCT02597348	90	France		Sep. 2015–Feb. 2027	Active, not recruiting
	SECA-III	NCT03494946	30	Norway		Dec. 2016–Jan. 2027	Recruiting
	SOULMATE	NCT04161092	45	Sweden		Dec. 2020–Dec. 2029	Recruiting
	EXCALIBUR1	NCT04898504	45	Norway		Aug. 2021–May. 2026	Active, not recruiting
Single arm DDLT	COLT	NCT03803436	22	Italy	Compared with the TRIPLETE trial (NCT03231722)	Jan. 2019–Jan. 2024	Recruiting
	MELODIC	NCT04870879	18	Italy		Oct. 2020–Oct. 2025	Recruiting
	TRANSMETIR	NCT04616495	30	Spain		Sep. 2021–Sep. 2028	Recruiting
		NCT05185245	20	Italy		Apr.2021–Mar.2030	Recruiting
		NCT05398380	35	Spain		Jan. 2021–Dec. 2026	Recruiting
		NCT04742621	20	USA		Sep. 2020–Jul. 2035	Recruiting
Single arm LDLT	Toronto Protocol	NCT02864485	20	Canada	Compared with subgroup dropout due to non-cancer-related reason	Aug. 2016–Dec. 2030	Recruiting
	LIVERT(W)OHEAL	NCT03488953	40	Germany	RAPID (LDLT)	Apr. 2018–Dec. 2027	Recruiting
		NCT05248581	50	USA		Aug. 2019–Aug. 2027	Recruiting
		NCT04874259	20	Korea		Aug. 2022–May 2026	Recruiting
		NCT05175092	20	Korea		Nov. 2023–Dec. 2030	Withdrawn
	LIVERMORE	NCT05186116	25	Italy		Jan. 2022–Jan. 2032	Recruiting
		jRCT1050230053	23	Japan		Dec. 2023–Nov. 2028	Recruiting
	RAPID	NCT02215889	20	Norway	RAPID (LDLT)	Jun. 2014–Jun. 2028	Recruiting
Others	RAPID-Padova	NCT04865471	18	Italy	RAPID (LDLT or DDLT)	Oct. 2020–Oct. 2025	Recruiting
		NCT06069960	40	China	RAPID (LDLT or DDLT)	Oct. 2023–Jun. 2027	Not yet recruiting
	CLEAR Trial	NCT06698146	80	USA	LDLT (right or left graft), LDLT (RAPID), DDLT (RAID), DDLT (whole liver)	Dec. 2024–Jan. 2035	Not yet recruiting
	LTLR-LC	NCT05750329	30	China	RAPID	Aug. 2023–Dec. 2026	Not yet recruiting

The success of this trial is attributed to stricter patient selection criteria compared with the Norwegian SECA study. Although the recurrence rate after transplantation was high at 74%, > 50% of the recurrences were limited to the lungs, and 46% of the recurrences were amenable to local therapies. The secondary 5-year recurrence-free survival rate after treatment was 36%, and no significant adverse events associated with chemotherapy after liver transplantation were reported. This report has increased awareness among oncologists about the potential utility of liver transplantation for the treatment of unresectable colorectal cancer with liver metastasis [42].

Although most clinical trials involve DDLT, a report from 3 North American centers in 2022 reported 10 cases of LDLT [43]. None of the donors experienced major complications, and the recipients showed excellent outcomes, with 1.5-year overall survival and recurrence-free survival rates of 100% and 62%, respectively. Currently, several studies focusing on LDLT are being conducted worldwide [44], raising expectations for the development of novel therapeutic strategies in regions with limited access to deceased donor organs. 

### Potential expansion of indications for resectable patients

A retrospective analysis conducted by a Norwegian group compared 53 cases of liver resection after portal vein embolization with 50 cases of liver transplantation with the same degree of disease progression. For left-sided colon cancer, the liver transplantation group had better outcomes than the liver resection group [45]. One potential factor influencing this difference is that left-sided colon cancer tends to involve fewer cases of lymph node metastasis than right-sided colon cancer, which may have affected the results. Furthermore, there have been reports indicating that, when compared based on metabolic tumor volume assessed by 18F-FDG PET/CT, liver transplantation is associated with a more favorable prognosis than liver resection [46]. liver transplantation may offer better outcomes than liver resection under certain conditions. Currently, there is a lack of sufficient evidence supporting the benefits of liver transplantation for resectable cases, and with the ongoing shortage of organ donors, it remains difficult to reach a social consensus regarding liver transplantation in resectable cases. However, depending on future research progress, it is possible that the indications for liver transplantation may expand.

At a limited number of specialized centers, super-extended surgeries—such as ex vivo liver resection with autotransplantation and ante-situm liver resection—have been employed to achieve R0 resection. However, even in cases where R0 resection is accomplished, the outcomes have not been entirely satisfactory. According to a systematic review encompassing super-extended resections for tumors including but not limited to colorectal liver metastases, the 90-day mortality rate was reported as 7.3%, with 1-year and 5-year overall survival rates of 72.3% and 23.4%, respectively. Recurrence rates at 1 and 5 years were 38.7% and 86.1%, respectively [47]. Reports from single-center experiences have demonstrated even less favorable outcomes, with a 90-day mortality rate of 28.6% and 1- and 5-year survival rates of 57.1% and 9.5%, respectively [48], which can hardly be considered satisfactory. While achieving R0 resection may offer a prognostic advantage over non-resection, the long-term outcomes seems to remain inferior to those of liver transplantation, and such an approach is unlikely to be established as a standard treatment modality.

## Future prospects

### Expansion of LDLT, use of marginal donor livers, and the introduction of machine perfusion technology

Given the global shortage of donor livers, patients with liver diseases with low evidence of transplantation eligibility face challenges in organ allocation. Consequently, strategies for the effective use of marginal donor livers are being explored. Liver transplantation for perihilar cholangiocarcinoma or CRLM has not yet been established as a standard treatment, and access to the donor organ remains limited under the current circumstances. One potential solution is LDLT. The advantages of this approach include the ability to schedule transplantation in advance and a short cold ischemia time; however, the disadvantages include risks to the donor, smaller graft size, and increased technical difficulty of the procedure. A second potential solution is the introduction of machine perfusion technology [49]. This approach can yield grafts that were previously deemed unsuitable for transplantation. Despite remaining challenges related to equipment investment and technical expertise, this is anticipated to be a future effective strategy.

### Use of immune checkpoint inhibitors after liver transplantation

Although immune checkpoint inhibitors (ICIs) have become widely available in recent years, their administration prior to liver transplantation increases the risk of rejection because immunosuppressive agents are essential after organ transplantation. Several reports have documented liver transplantation following downstaging with ICIs for hepatocellular carcinoma. However, the outcomes have been highly variable, ranging from cases of uneventful post-transplantation courses [50, 51] to instances of rapid and severe rejection leading to early postoperative mortality [52, 53]. Although the washout period for ICIs is considered crucial, existing reports have shown significant variability in treatment duration and washout intervals, making it difficult to draw definitive conclusions [51]. Regarding the use of ICIs for cholangiocarcinoma, a phase III study (TOPAZ-1) reported that durvalumab in combination with gemcitabine and cisplatin was more effective than a placebo in combination with gemcitabine and cisplatin [54, 55]. Similarly, the addition of pembrolizumab to gemcitabine and cisplatin was evaluated in the KEYNOTE-966 trial [56]. However, the risk assessment of liver transplantation following ICI treatment remains insufficient, so further evidence is needed. In the context of CRLM, the use of ICIs remains limited to anti-PD-L1 therapy for microsatellite instability-high (MSI-high) tumors. However, with ongoing advances in chemotherapy, similar challenges may emerge in the future.

### Management of immunosuppressive medicine

Liver transplantation for hepatocellular carcinoma improves overall survival and recurrence-free survival with the postoperative use of mammalian target of rapamycin (mTOR) inhibitors while reducing the risk of nephrotoxicity [57]. In addition to their immunosuppressive effects, mTOR inhibitors are expected to exert antitumor effects. However, their antitumor effects in cholangiocarcinoma and colorectal cancer remain unclear. Although the guidelines recommend considering their use in the absence of specific contraindications [33], long-term follow-up is essential.

## Conclusions

**Fig. 1:**
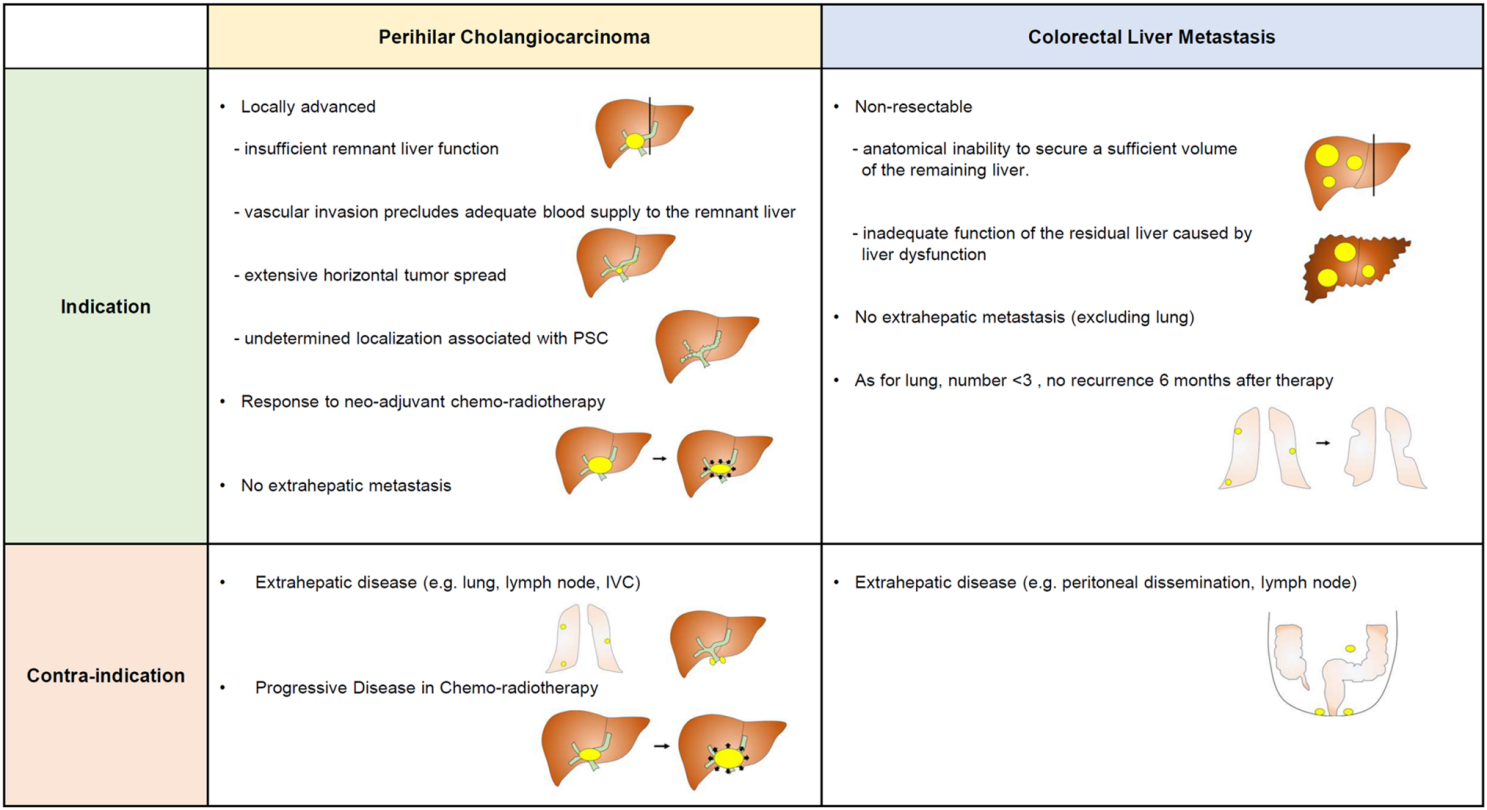
The summary of the indication and contra-indication for liver transplantation for unresectable cholangiocarcinoma and colorectal cancer with liver metastases

The indication and contra-indication for liver transplantation for unresectable cholangiocarcinoma and colorectal cancer with liver metastases are summarized in Fig. 1. These treatments must be considered part of a multidisciplinary treatment approach that includes chemotherapy, radiotherapy, and other modalities. This requires collaboration among not only transplant surgeons, but also colorectal, hepatobiliary-pancreatic, medical, and radiation oncologists [40]. With advancements in techniques across these specialties, it is hoped that new treatment options will become available to patients who have previously been deemed incurable.
